# Site-specific gene expression patterns in oral cancer

**DOI:** 10.1186/s13005-017-0138-0

**Published:** 2017-05-10

**Authors:** Gesche Frohwitter, Horst Buerger, Eberhard Korsching, Paul J. van Diest, Johannes Kleinheinz, Thomas Fillies

**Affiliations:** 1 Institute of Pathology, Husener Str. 46a, 33098 Paderborn, Höxter Germany; 2 Institute of Pathology, University of Utrecht, Utrecht, The Netherlands; 3 Institute of Bioinformatics, University of Muenster, Muenster, Germany; 4 Department of Cranio- and Maxillofacial Surgery, University Hospital Muenster, Muenster, Germany; 5 Department of Cranio- and Maxillofacial Surgery, Marienhospital Stuttgart, Stuttgart, Germany

**Keywords:** Squamous cell carcinoma, Oral cavity, Location, Tumour biology

## Abstract

**Background:**

Squamous cell carcinomas (SCCs) are the most prevalent malignant tumours within the head and neck. Evidence exists that distinct genes are differentially regulated in SCCs of the oral cavity compared to other head and neck regions. Given this background, the aim of this study was to investigate whether such tumour site-specific gene expression can also be observed in different localizations within the oral cavity.

**Methods:**

Using tissue microarrays (TMAs), we investigated 76 SCCs of the floor of the mouth, 49 SCCs of the tongue and 68 SCCs of other anatomic regions within the oral cavity. The expression of 17 genes involved in cell cycle and growth control (p16, p21, p27, p53, cyclin D1, EGFR, c-kit, bcl-6), cell adhesion (alpha-, beta-, and gamma-catenin), and apoptosis/stress response genes (Hif-1-alpha, Glut 1, CA IX, caspase, hsp70, XIAP) were investigated by means of immunohistochemistry. The data were subjected to chi^2^, interdependency and Kaplan-Meier analysis.

**Results:**

Our study suggests a remote difference in the site-specific gene expression patterns of oral cancer. X-linked inhibitor of apoptosis (XIAP) showed a significantly higher expression (*p* <0.05) in SCCs of the floor of the mouth compared to SCCs of the tongue and other locations within the oral cavity. The increased XIAP expression was further associated with significantly decreased overall survival in all cases of SCCs of the oral cavity (*p* <0.05). Expression levels of p53, CA IX, beta-catenin, Hif-1-alpha, and c-kit were also observed to be inversely related between SCCs of the floor of the mouth and those of the tongue respectively, although these differences did not reach statistical significance. Overall and event-free survival did not differ in patients with T1/T2/N0 SCCs according to tumour localization.

**Conclusion:**

In summary, the protein expression patterns of SCCs of the oral cavity suggest the existence of a molecular and morphological spectrum of SCCs in the oral cavity. In particular the expression pattern of XIAP indicates distinct gene expression patterns between carcinomas of the floor of the mouth and oral tongue cancer. Further studies are needed to identify possible tumour site-specific factors that influence patient prognosis and management.

## Background

Malignant oral neoplasms are a heterogeneous category of cancer, which are dominated by squamous cell carcinomas (SCCs) [1]. The incidence of oral SCCs has been continuously on the rise, now underlined by representing the tenth most common type of cancer and accounting for 260,000 new cases and 128,000 deaths per year worldwide [2]. To date, surgery has been the benchmark strategy for the primary treatment of oral SCCs, involving radical tumour resection, neck dissection, and plastic reconstructive surgery. The extent of surgical therapy required is determined by the spread of the tumour according to TNM-classification after staging as well as for patients with physical and mental strain. Supplemental therapy such as radiation and chemotherapy plays an important role, especially in T3 and T4 tumours as well as in cases with positive lymph nodes, relapses, and palliative situations. Long-term outcome, even in small tumours without histopathologically diagnosed lymph node involvement is highly unpredictable, leading to an overall 5-year-survival rate of 50% without any change for the last few decades [1, 3]. It is therefore important to shed light on the molecular behaviour of cells, proteins, and enzymes involved in oral SCC development and progression to be able to detect patients with highly aggressive cancer and initiate appropriate therapy.

Several studies have reported specific metastatic pathways according to tumour localization and different responses to radiation therapy depending on the anatomical site [4–6]. Belbin et al. showed that specific biological mechanisms underlying tumour aggressiveness are heavily influenced by the site of the primary tumour [7]. Furthermore, it has been reported that oral SCCs of different anatomic locations of the oral cavity express an abnormal amount of cell cycle regulation proteins [8]. We therefore hypothesized that there is a difference in the pattern of molecular tumour development according to the anatomic site. Substantiating this hypothesis was the purpose of our investigation.

## Methods

### Patients

The tested samples were procured by the Institute of Pathology, University of Muenster, Germany. A total of 193 formalin-fixed, paraffin-embedded archival cancer tissue samples of oral SCCs were tested. Details on the clinical procedures and pathological methods of the tumour series are provided in previous publications [9–11]. As shown in Table 1, the series was composed of 193 patients (39 females, 154 males) with a mean age of 59 years (range 31-90 years). The TNM classification of the tumour samples is based on the histopathological tumour evaluation (pTNM). According to the TNM system, the post-surgical classification revealed 96 T1 tumours, 82 T2 tumours, and 15 T3/4 tumours, 136 patients had a negative (N0) locoregional nodal status, whereas 57 patients showed positive (N > 0) locoregional lymph nodes. All patients eligible for the study received continuous follow-up examinations for 4-181 months, the data from patients that failed to regularly attend the follow-up program were not considered after the last regular examination. The time of survival was defined as the period from the surgery day to the date of histologically proven recurrent or metastatic disease, or to the day of death, or to the day of the last follow-up care (60 months post-surgery) [9–11].

**Table 1:** Tumour patient collective and clinicopathological features of the tumour samples evaluated in the study

Age at diagnosis (mean)	59 years (rage 31–90 years)
Sex	
Female	39
Male	154
T stage	
T1	96
T2	82
T3-T4	15
N stage	
Lymph node negative	136
Lymph node positive	57
Grading	
G1	44
G2	126
G3	23
Recurrent disease	
positive	66
negative	127
Localization	
Floor of the mouth	76
Tongue	49
Other	68

### Immunohistochemistry

A total of 193 cancer tissues samples were examined for the expression of p16, p21, p27, p53, cyclin D1, EGFR, c-kit, bcl-6, alpha-, beta-, and gamma-catenin, Hif-1-alpha, Glut 1, CA IX, caspase, hsp70, and XIAP.

To ensure identical conditions for the investigation of all tumour specimens, we used tissue microarrays (TMAs) and immunohistochemistry. As described in earlier publications, all TMAs were constructed under a standard protocol [12].

For the preparation of the TMA, each donor block was used to supply the new acceptor block with two punch biopsies measuring 0.6 mm in diameter. The samples where taken at the tumour margin to ensure consideration of the tumour front in histopathological analysis. Therefore, a special TMA construction tool was used according to the guidelines of Beecher Instruments, New Jersey, USA [12].

The immunohistochemistry was carried out on 4-μm-thick sections. The source of the antibodies, clones, dilutions and the antigen retrieval are shown in Table 2. The peroxidase system contained methanol with 0.3% hydrogen peroxide (Walter-CMP GmbH & Co. KG) and had an exposure time of 30 min. The expression patterns were evaluated in a semi-quantitative manner.

**Table 2:** List of antibodies, source, clone, dilution and antigen retrieval applied in the study

Antibody	Supplier	Catalogue Number	Clone	Mono/Polyclonal	Species	Dilution	Antigen Retrieval
p16	CINTec/Roche	9517	E6H4	Mono	Mouse	KIT-	Citrate buffer pH6.0
p21	Merck Millipore	05–655	CP 74	Mono	Mouse	1:500	Citrate buffer pH6.0
p27	BD TL	610241	57/Kip1/p27	Mono	Mouse	1:1000	Citrate buffer pH6.0
p53	Dako	M7001	DO-7	Mono	Mouse	1:100	EDTA pH8.0
Hif-1-alpha	BD TL	610958	54/HIF-1a	Mono	Mouse	1:50	EDTA pH8.0
Glut 1	Dako	M 7211	Clone A 35	Mono	Mouse	1:40	EDTA ph8.0
Ca IX	Abcam	ab128883	-	Poly	Rabbit	1:1000	Citrate buffer pH6.0
XIAP	BD TL	610716	28/hILP/XIAP	Mono	Mouse	1:50	Citrate buffer pH6.0
Hsp 70	Invitrogen	33–3800	MB-H1	Mono	Mouse	1:40	Citrate buffer pH6.0
a-Catenin	BD TL	610194	5/a-Catenin	Mono	Moue	1:250	EDTA pH8.0
b-Catenin	BD TL	610153	14/beta-Catenin	Mono	Mouse	1:1000	EDTA pH8.0
g-Catenin	BD TL	610253	15/g-Catenin	Mono	Mouse	1:1500	EDTA pH8.0
BCL-6	Dako	M7211	PG-B6p	Mono	Mouse	1:50	Citrate buffer pH6.0
Caspase 3	Invitrogen	35-1600Z	43191	Mono	Mouse	1:100	Citrate buffer pH6.0
C-kit	Dako	A 4502	-	Poly	Rabbit	1:200	Citrate buffer pH6.0
Cyclin D1	Novocastra	NCL-L-Cyclin D1-GM	P2D11F11	Mono	Mouse	1:20	EDTA pH8.0
EGFR	Dako	K 1492	pharmDX-Kit	Mono	Mouse	KIT	-

### Scoring

The thresholds for most markers have been described previously [9, 10, 13]. The expression of cytokeratins was measured by the rate of positively stained cells in each core. CK19 (0%, no expression; 1-50%, moderate expression; >50%, high expression) and in two groups for CK 1, 5/6, 8/18, 10 (0%, no expression; ≥ 1%, positive expression). The percentage value of two biopsies from one tumour was evaluated. Irrespectively of the number of stained tumour cells cytoplasmatic expression of XIAP, Caspase 3 and Hsp 70 was graded as negative or positive (intermediate to strong expression). CAIX, GLUT 1 and p16/21/27 were rated in two grades (no expression < 1%, ≥ 1% positive expression). The following markers were graded in three groups: HIF-1a (<1% no expression, low expression ≥ 1% - < 5%, high expression ≥ 5%), BCL-6, a-Catenin (0–15% no expression, 16–50% low expression, 85–100% positive expression), b-catenin and g-catenin (0–15% no expression 16–50% low expression, 51–100% high expression). EGFR, Cyclin D1 and C-kit were measured as follows 0–15% no expression, 16–50% low expression and 85–100% positive expression. p53 was rated as no expression < 5%, ≥ 5%-50% low expression and ≥50% high positive expression.

### Statistical analysis

Statistical analysis related to clinicopathological factors was performed using chi^2^ analysis and Kaplan-Meier analysis. A statistical test on differences between the slope of the regression lines was performed (Table 3). The test is based on two linear models: a) y ~ b0 + b1x + b2g (null model) and b) y ~ b0 + b1x + b2g + b3xg (alternate model) where b_i_ are the model coefficients, g the grouping factor and xg the dependency term. Taking the ANOVA/F test on the fit of the two models gives significant differences in our case by assuming alpha values of smaller than 0.05.

**Table 3:** Test on significant different slope

	Floor of the mouth	Tongue	Other
Floor of the mouth	0	0	0
Tongue	6.9e-07	0	0
Other	3.1e-02	0.11	0
Tongue	1.2e-07	0	0
Other	5.8e-03	0.025	0

The immunohistochemical data of the TMA tissue was additionally evaluated by an interdependency analysis [14, 15]. This approach provides the possibility of retrieving the strength of support of a set of molecular markers to a certain anatomic region [16]. Given those differences it can be concluded that the present region owns slightly different molecular regulation schemes. The detailed description of this approach and its application in a clinical setting using TMA data has been provided previously [17–19].

## Results

**Fig. 1:**
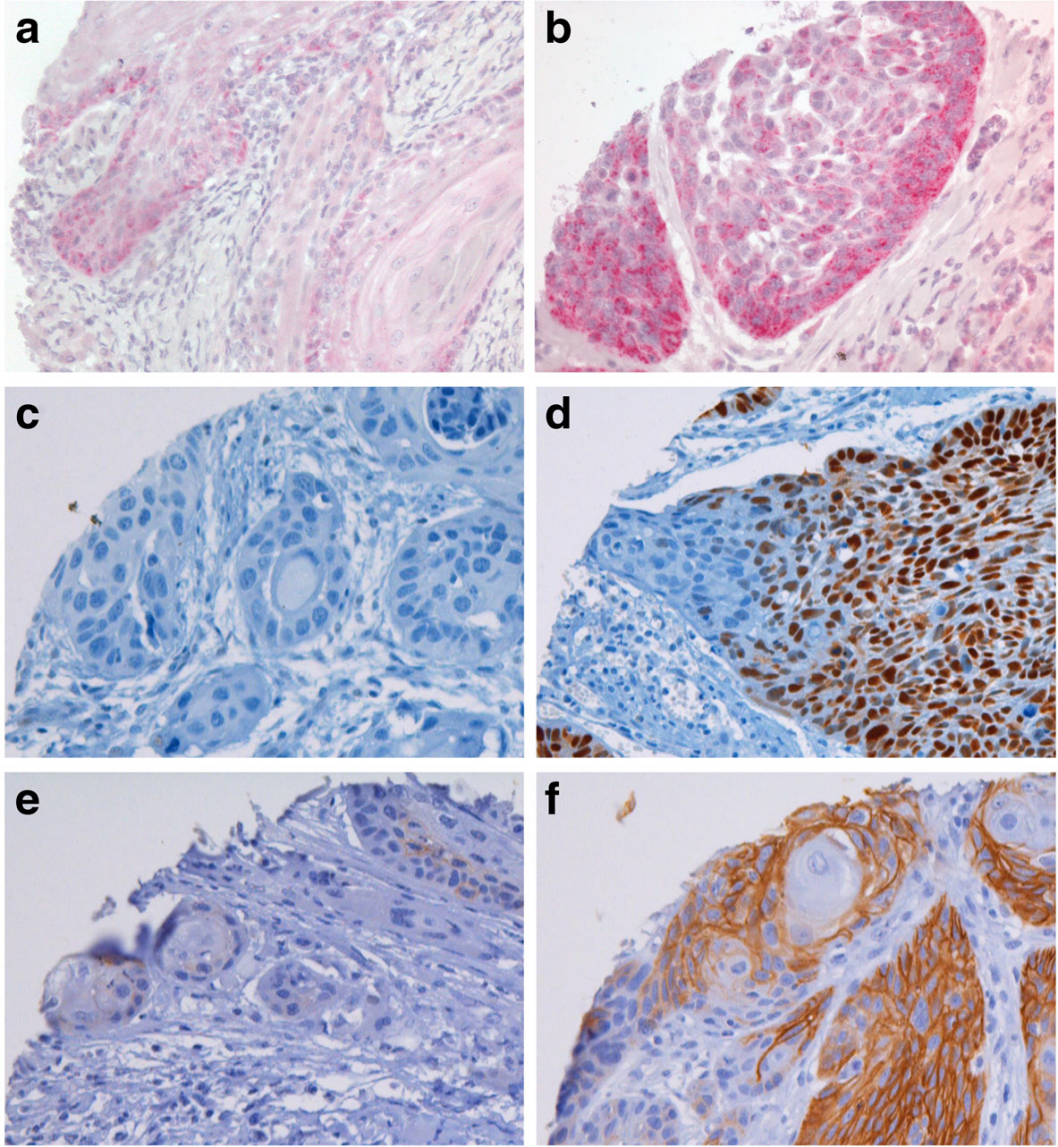
Examples of positive immunohistochemical staining of oral squamous cell carcinomas with XIAP, p53 and CAIX antibodies. **a** weak XIAP expression, **b** strong XIAP expression, **c** weak p53 expression. **d** strong p53 expression, **e** weak CAIX expression, **f** strong CAIX expression

The results of the immunohistochemical expression patterns according to different tumour locations are summarized in Table 4. Examples of positive immunohistochemical stainings of oral SSC with XIAP, p53 and CAIX antibodies are given in Fig. 1.

**Table 4:** Expression profile of antibodies at different tumour localizations used in the study and measured in per cent/*p* value/*r* value

Antibody	Floor of the mouth positive expression	Tongue positive expression	Other positive expression
p16	22.4/0.67/0.03	18.6/0.64/-0.03	22.9/0.72/0.03
p21	70.6/0.82/-0.02	73.8/0.76/0.02	75.5/0.61/0.04
p27	19.7/0.59/0.04	20/0.95/0	18,4/0.93/0.01
p53	52,9/0.21/0.09	85.7/0.46/-0.05	55/0.32/-0.07
Hif-1-alpha	63.2/0.27/0.08	44.9/0.11/-0.12	59.1/0.7/0.03
Glut 1	90.0/0.28/0.08	85.7/0.3/-0.07	96,9/0.84/-0.01
Ca IX	22.1/0.2/-0.09	33.3/0.17/0.1	27/1/0
XIAP	30.0/0.01/0.18	13.2/0.31/-0.07	12.5/0.04/0.15
Hsp 70	10.8/0.89/-0.01	10.3/0.61/-0.04	10.9/0.34/-0.07
a-Catenin	64.8/0.71/-0.03	65.9/0.91/0.01	65.7/0.91/-0.01
b-Catenin	84.5/0.78/-0.02	86.7/0.09/0.12	82.1/0.08/-0.13
g-Catenin	66.7/0.98/0	64.3/0.84/0.02	63.6/0.89/0.01
BCL-6	25.8/0.63/0.03	18.2/0.58/-0.04	19.1/0.84/-0.01
Caspase 3	32.8/0.07/0.07	21.1/0.3/-0.03	22/0.3/-0.01
C-kit	16.2/0.49/0.05	6.8/0.36/-0.07	15.4/0.26/0.08
Cyclin D1	51,5/0.63/-0.03	45.5/0.44/0.06	50/0.72/-0.03
EGFR	81.2/0.91/0.01	68.2/0.56/-0.04	73.5/0.85/0.01

**Fig. 2:**
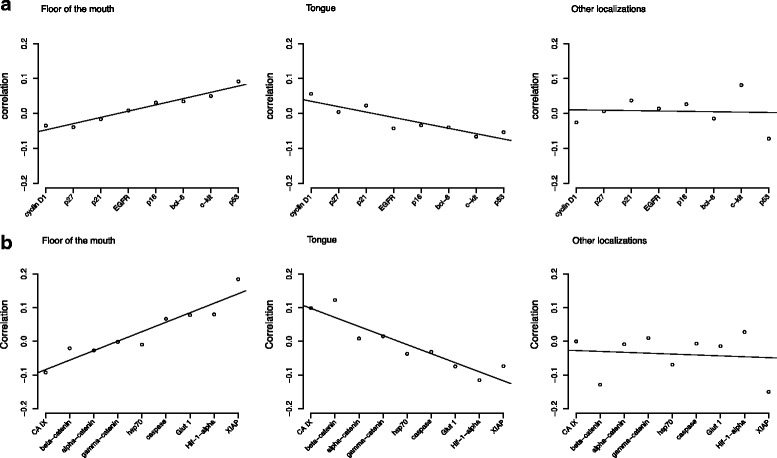
Regression curves of the evaluated tumour samples examined by permutation analysis. **a** Protein expression of different anatomical subsites analysed according to cell cycle and growth control regulation proteins. **b** Protein expression of different anatomical subsites analysed according to genes involved in cellular stress responses, apoptosis and cell adhesion

The results of the interdependency analysis for the different expression patterns of SCCs in various locations of the oral cavity are shown in Fig. 2. Two different test sets have been generated, containing 9 and 8 test markers, respectively. The correlation between the test marker (x-axis) and the location surrogate marker is shown on the y-axis. The first test set included cell cycle control proteins and two growth factor receptors. In the second set genes involved in cellular stress responses, apoptosis, and cell adhesion were investigated.

In the first marker set only minor differences between the different tumour localizations could be observed (Fig. 2a). SCCs of the floor of the mouth and of the tongue showed opposing regression curves. In SCCs of the floor of the mouth positive correlation coefficients were observed for p53 and c-kit, whereas the expression of these protein showed a negative correlation in SCCs of the tongue. A similar but inverse pattern was revealed for cyclin D1 expression. The regression curve for SCCs of various other localizations within the oral cavity did not reveal any significant regression trends.

**Fig. 3:**
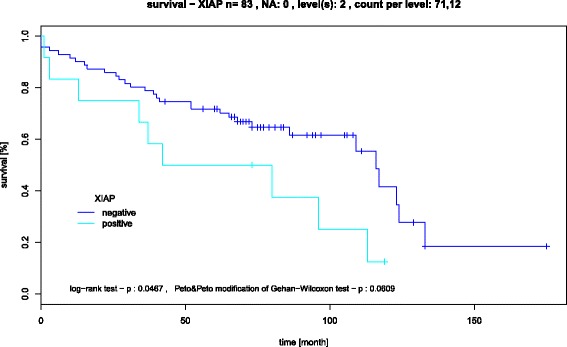
Kaplan-Meier survival curve, showing that the expression of XIAP was associated with an unfavourable prognosis in all SCC’s of the oral cavity (*p* <0,05)

The second test set (Fig. 2b) showed more prominent differences in the behaviour of the test markers. HIF-1-alpha and XIAP had a remarkable and different regulatory role in SCCs of the floor of the mouth and tongue, whereas the appearance of XIAP in other tumour localizations had no impact (*p* <0.05), Fig. 2b. Furthermore, the expression of XIAP was associated with a poor prognosis in all SCCs of the oral cavity (*p* <0.05), as shown in Fig. 3.

As demonstrated in Fig. 2b, SCCs of the tongue showed positive expression of CA IX and beta-catenin. The regression curve for the SCCs of other localizations within the oral cavity did not reveal to differentiate gene expression patterns in relation to tumour localization.

Table 3 shows the test on significant different slope.

Table 4 shows the expression profile of antibodies at different tumour localizations measured in per cent. Taking XIAP as an example, the positive staining results for the floor of the mouth (30%) in comparison those for the oral tongue (13.2%) and other tumour localizations (12.5%) were consistent with the regression curves shown in Fig. 2b.

In summary, the opposing trends of the regression curves for SCCs of the floor of the mouth and of the tongue indicate a slightly different regulatory role of XIAP as a tumour marker.

**Fig. 4:**
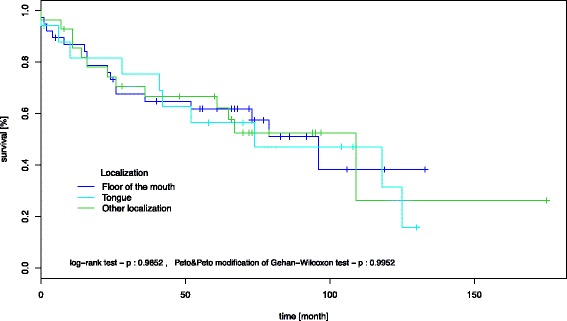
Kaplan-Meier survival curve, showing that overall and event-free survival did not differ in patients with T1/T2/N0 SCCs according to tumour localization

However, overall and event-free survival did not differ in patients with T1/T2/N0 SCCs according to tumour localization (Fig. 4).

## Discussion

SCCs of the oral cavity account for more than 90% of all malignant neoplasms in this anatomic region. Apart from Asian, countries where buccal oral SCCs rank first on the list of anatomical sites, in Western countries, the oral tongue is most frequently affected (40–50%) followed by the floor of the mouth [3, 20]. These differences appear to be mainly due to various exogenous risk factors rather than an intrinsic molecular ethnic background [3, 21]. Hence, the data suggest the possibility of the existence of multiple lines of evolution of oral SCCs according to their anatomic localization and the presence of respective risk factors. From a histomorphological point of view, SCCs of the head and neck region, including the oral cavity, are typed and graded similarly suggesting related underlying tumour biology. However, recent evidence has demonstrated SCCs of the oral cavity and the head and neck region might actually be different tumour entities at the molecular level [22, 23].

Belbin at al. detected altered gene expression levels at different anatomic sites in the head and neck by examining the whole RNA sequence of 45 head and neck SCCs compared to samples of a healthy control group. Out of the wide range of genes identified, Belbin et al. extracted TGF β, IL 1, and matrix metalloproteinases as typical of oral SCCs, PCK, IL 8, and FGFR 1 as characteristic genes of oropharyngeal SCCs, and IL 6, p53, and PRLR as representative of hypopharyngeal and laryngeal SCCs, suggesting distinctive hallmarks for each anatomical subsite in head and neck SCCs [7]. In 2011, Boldrup et al. emphasized the importance of differentiating the anatomical subsites as well as the histological mucosa conditions to sufficiently evaluate the histomorphological patterns of head and neck cancer [8]. Furthermore, the outcome of radiation therapy in advanced disease has been suggested to be associated with the anatomic location of the tumour [6].

Our own results as well as those described above support our theory that tumour development in the head and neck area can be evaluated as a single interlocked path affected by exposure to carcinogenic substances. However, the individual tumour growth patterns and hence the highly variable therapy responses observed may be influenced by other factors.

Based on these observations, we evaluated whether SCCs of different anatomic localizations within the oral cavity might also differ with respect to their molecular background. The molecular biology of malignant tumours often determines the clinical behaviour and long-term outcome. With our first approach, we were able to show that the overall and event-free survival did not differ in patients with T1/T2/N0 SCCs according to tumour localization (Fig. 4).

Using the TMA technique and a set of 17 different antibodies, we were able to show that different anatomic localizations within the oral cavity seem to be associated with slightly different molecular expression patterns. TMA is a profound method to evaluate immunohistochemical patterns in large numbers of tumour samples [12, 16]. Even though the method might be questionable when evaluating heterogeneous tissue or a single histopathological sample, the size of our patient collective (*n* = 193) as well as the statistical methods applied spares the idea of non-significant punch biopsies [15]. In particular SCCs of the floor of the mouth and the tongue showed different protein regulation patterns. For example, XIAP was strongly expressed in carcinomas of the floor of the mouth, an inverse result could be observed in SCCs of the tongue (Fig. 2b). XIAP is known as a member of the inhibitors of apoptosis family that compromises eight proteins preventing caspase activation. Furthermore, XIAP can affect initiator and effector caspases, and is capable of inhibiting the intra- and extramitochodrial apoptotic pathway [24]. The suppression of caspase 3, 7, and 9 activation favours tumour growth and also strengthens the resistance of tumour cells against the effects of cisplatin-based chemotherapy in advanced oral SCCs and oesophageal SCCs [25, 26]. Hence, XIAP expression contributes to a more resistant tumour with a lower response to adjuvant radiation therapy. However, due to the diversity of genes involved in tumour development and progression, it cannot be conclusively stated, that carcinomas of the floor of the mouth behave more aggressively than tongue carcinomas (Fig. 4). Nevertheless, one has to keep in mind, that the expression of XIAP without any relation to the tumour site did result in a decreased overall survival (Fig. 3).

Similar findings and tendencies could be observed for the expression levels of p53, CA IX, beta-catenin, Hif-1-alpha, and c-kit in both localizations.

Hypoxia inducible factor (HIF) is a heterodimer protein consisting of a three-part alpha subunit and a single beta subunit. In hypoxemia, induced cell stress, Hif-1-alpha functions as a transcription factor. As oxygen levels decrease, the alpha subunit accumulates with the beta subunit, transfers to the nucleus, and activates the hypoxia-responsive element that operates as a transcription factor. The upregulation of Glut 1, VEGFR, CA IX, erythropoietin, heat shock proteins, and other cell growth factors affect protein expression and activation. This cascade is involved in the differentiation of embryonic stem cells, bones, blood vessels, and organs as well as in tumour cells with underlining similarities in growth habits, leading to fast tumour progression [27–33]. Again, these findings are not able to confirm the extent of tumour aggressiveness in relation to its localization, but nevertheless point towards a complex pattern of gene interaction that varies even with respect to the anatomical subsite.

The other markers also showed globally differing regulation patterns but with lower impact. The interdependency analysis, which is a statistical tool used to reveal small differences in regulatory pathways, indicated that there are different underlying molecular mechanisms in SCCs of the floor of the mouth and the tongue. The regression curves showed an almost antagonistic protein expression profile between floor of the mouth cancer and oral tongue cancer. However, it has to be stated that the chi^2^ analysis only showed statistical significance for the differential expression of XIAP.

Using interdependency analysis in invasive breast cancer the existence of a number of independent, parallel progression pathways was identified [34]. Therefore, we cannot conclusively interpret the slightly opposing regression curves in SCCs of the floor of the mouth and the tongue, as well as the other anatomic sites, as clear evidence for multiple, independent progression pathways in SCCs of the oral cavity. Instead, we consider that our results point to a molecular and morphological spectrum of SCCs, with a possible influence of so far unknown site-specific factors on commonly shared tumour biological mechanisms. Further research is required to assess the importance of molecular site-specific tumour patterns in practice.

## Conclusion

In summary, we analysed 193 SCCs with a focus on 17 different protein expression patterns in relation to the anatomical location within the oral cavity using a sophisticated biomathematical algorithm. Our results point towards a wide molecular spectrum of SCCs in the oral cavity. Even though the carcinomas showed a large range of protein expression, only minimal site-specific protein mechanisms could be detected that could potentially reflect the different clinical behaviours of SSCs within the oral cavity. For T1/T2/N0 tumours no significant difference in tumour site-specific survival could be seen. Further studies are needed to define possible tumour site-specific factors with relevance for patient prognosis and management.

## Abbreviations

Bcl-6: B-cell lymphoma-6 cell adhesion
CA IX: Carbonic anhydrase 9
C-kit: Mast/stem cell growth factor receptor
EGFR: Epithelial growth factor receptor
Glut 1: Glucose transporter 1
Hif-1-alpha: Hypoxia inducible factor 1-alpha
Hsp70: Heat shock protein 70
SCCs: Squamous cell carcinomas
TMAs: Tissue mircoarrays
XIAP: X-linked inhibitor of apoptosis protein
